# Poisoning from the Use of Tobacco Enema

**Published:** 1842-01

**Authors:** 


*Poisoning from the use of a Tobacco Enema.*—A strong man, aged 55, who had for some time labored under dysuria, from enlarged prostate, and more recently had suffered from ascarides in the rectum, received an enema, made by infusing 12 grains of tobacco in 12 oz. of water. Seven or eight minutes afterwards he appeared in a slight stupor, had headache, and unusual paleness of the face. He complained of pain in the belly; his answers to questions were not clear. Two purgative injections were given, sinapisms applied, stimulants given and a small quantity of blood (three pallettes) taken. The paleness increased, coma and convulsions followed; extreme prostration, and finally death closed the scene.—*Rev. Med.*

*Remarks.*—We are induced to copy this case, that we may impress, if possible, our readers with our own conviction of the extreme danger from tobacco enemata. Early in professional life, a case came to our knowledge, which has ever served to keep us from this remedy. A young sailor, healthy and robust, was admitted into the Pennsylvania Hospital with strangulated hernia; the taxis, &c., were tried in vain. An operation was resolved on; somebody suggested a tobacco enema; it was tried, and in less than two hours the poor fellow died, prostrated, poisoned by this unlucky remedy. A method of using this remedy per anum, which was lately resorted to by our friend, Dr. Jno. R. McComb, may be attended with less risk than the enemeta. He enveloped a dose of tobacco in a fine linen rag, put it up the anus as a suppository, and as soon as the nausea was produced, removed the whole. This is probably an improvement, but even thus we should resort to tobacco only at the last extremity and then with fear and trembling.—*N. Y. Med Gaz.*

